# Machine learning-based assessment of storm surge in the New York metropolitan area

**DOI:** 10.1038/s41598-022-23627-6

**Published:** 2022-11-10

**Authors:** Mahmoud Ayyad, Muhammad R. Hajj, Reza Marsooli

**Affiliations:** Davidson Laboratory, Department of Civil, Environmental and Ocean Engineering, Stevens Institute of Technology, Hoboken, NJ 07030 USA

**Keywords:** Natural hazards, Climate-change mitigation

## Abstract

Storm surge generated from low-probability high-consequence tropical cyclones is a major flood hazard to the New York metropolitan area and its assessment requires a large number of storm scenarios. High-fidelity hydrodynamic numerical simulations can predict surge levels from storm scenarios. However, an accurate prediction requires a relatively fine computational grid, which is computationally expensive, especially when including wave effects. Towards alleviating the computational burden, Machine Learning models are developed to determine long-term average recurrence of flood levels induced by tropical cyclones in the New York metropolitan area. The models are trained and verified using a data set generated from physics-based hydrodynamic simulations to predict peak storm surge height, defined as the maximum induced water level due to wind stresses on the water surface and wave setup, at four coastal sites. In the generated data set, the number of low probability high-level storm surges was much smaller than the number of high probability low-level storm surges. This resulted in an imbalanced data set, a challenge that is addressed and resolved in this study. The results show that return period curves generated based on storm surge predictions from machine learning models are in good agreement with curves generated from high-fidelity hydrodynamic simulations, with the advantage that the machine learning model results are obtained in a fraction of the computational time required to run the simulations.

## Introduction

The New York metropolitan area, including New York City (NYC), Long Island, and the east coast of New Jersey (NJ), covers a region of narrow rivers, estuaries, islands, and sand barriers. Most of the region has an elevation that is less than 5 m above mean sea level^1^ which makes it vulnerable to storm surge flooding due to Tropical Cyclones (TCs). In the past 10 years, TCs Sandy in 2012 and Isaias in 2020 killed tens of people and damaged thousands of houses^2–4^. The damage to infrastructure caused interruptions to supplies of clean water, electricity, and transportation. These risks and damages are expected to become more pronounced in the future due to the expected increase in the intensity of TC, shift in their track, and potential change in their frequency in relation to climate change^5–19^.

Long-term mitigation and adaptation strategies to storm surge require quantifiable predictions of flood hazards under different scenarios while taking into consideration coastal development. These predictions are often presented in terms of a range of N-year return periods of a peak storm surge height defined as the height with 1/N percent chance of exceedance in any given year. In many coastal cities, a 10-year return period is used to assess impact of moderate storm surge scenarios that would cause temporary disruptions and minor damage to buildings. A 100-year return periods is used to assess the impact of extreme storm surge causing more significant damage over a broader coastal region. In countries and cities that are more prone to flooding, larger return periods are used to determine the design water level. In the Netherlands, 4000- and 10,000-year return periods are used for coastal flood defense works^20^. Because the peak storm surge increases with the return period, the higher return periods (low probability) correspond to higher storm surge (high-consequence event) while low return periods (high probability) correspond to lower storm surge (low-consequence event). For a reliable estimate of the N-year flood level one should consider $10 \times N \times f$ storm scenarios^21^ where *f* represents the annual storm frequency. Ayyad et al.^22^ estimated that 600,000 storm scenarios will be required to reliably predict 1000-year return period when taking uncertainties associated with climate change into consideration.

Because the number of historical storms is limited and could not account for future changes, numerical model simulations of synthetic storms are usually used to determine storm surge levels. The most used hydrodynamic models include Sea, Lake, and Overland Surges from Hurricanes (SLOSH)^23^ and the coupled Simulating WAves Nearshore and ADvanced CIRCulation^24^ (ADCIRC + SWAN) models^25,26^. The high computational cost, which is significantly increased for high-resolution (street-level) hydrodynamic simulations, hinders the ability to perform the number of simulations required to predict low-probability high-consequence events. Alternatively, data-driven surrogate models, trained with physics-based simulations, can be used to effectively reduce this computational cost. Ayyad et al.^22^ demonstrated that the Artificial Neural Network (ANN) technique can be effectively combined with physics-based simulations to reliably predict storm surge on an ideal coast that did not consider variations in coastal features or how resolution requirements can impact their generalized approach. Richardson et al.^27^, and Das et al.^28^ developed regression and data-based algorithms to predict storm surge height by calculating a similarity index from the TC parameters and match it with an existing TC database. Ruckert et al.^18^ discussed different examples of probabilistic projections of sea level rise^29–32^. Others used shallow ANN models to predict surge levels due to synthetic or historical Typhoon data^33–37^. Hashemi et al.^38^ and Lee et al.^39^ used ANN models to respectively predict maximum water elevation and storm surge height time series on the basis of synthetic TCs. Tiggeloven et al.^40^ used different ANN model architectures to predict hourly surge time series prediction of the global tide and surge model forced using atmospheric variables. Other studies used kriging methods to predict storm surge height time series^41–43^. Yousefi et al.^44^ used machine learning models to predict snow avalanches, and floods using climatic, topographic, and morphological factors as the input variables. The aforementioned surrogate models have few limitations. In most of these studies, the storm parameters were defined at their time of landfall or were assumed to remain constant as the TC tracks, which is not the case in a real-life application. Also, the models mostly used small data sets, less than 1100 storms, for training, validating and testing the surrogate models, which impacts the accurate prediction of low probability events that are of high consequence.

In this study, we demonstrate the use of Machine Learning (ML) models to predict low-probability peak storm surge height due to TCs. We perform different feature selection techniques to generate a perfect ML model. We avoid all the aforementioned limitations and address the challenge of modeling low-probability high-consequence events. A large data set of more than 10,000 synthetic TCs is used without the need to assume that the TC parameters remain constant along its track. The peak storm surge height is calculated from synthetic TCs using the coupled ADCIRC + SWAN models. Also, TC parameters at the time of landfall and 6 h before and after landfall are used as the ML input features. Implementing this approach to four study sites in the NY metropolitan area allows for identification of differences in model predictions associated with specific coastal features.

## Methods

### Data set generation

#### Hydrodynamic model

The coupled ADCIRC + SWAN model is used to simulate storm surge height including the effects of wave-current interactions and variations in water depth. **ADCIRC**^24,45^ is a finite-element hydrodynamic model used to simulate free surface circulation and transport problems. In this study we used the two-dimensional depth-integrated version, referred as ADCIRC-2DDI. It solves the generalized wave-continuity and vertically-integrated momentum equation to calculate the free water surface elevation and depth-averaged velocities, respectively. In the current simulations, ADCIRC’s Holland hurricane model^46,47^ is used to calculate the wind and pressure fields. **SWAN**^25^ is a finite difference model used to simulate the generation, propagation, and dissipation of surface gravity waves in deep ocean and coastal waters. SWAN solves the wave action balance spectrum equation to calculate the phase-averaged wave characteristics, e.g., significant wave height. The source term in the governing equation includes input energy from wind, dissipation by bottom friction, wave breaking, and nonlinear wave-wave interactions^48^. **ADCIRC + SWAN**^49^ is a coupled model that is widely used by academia, industry, and state/federal government agencies (e.g., Dietrich et al.^49^, Xie et al.^50^, Marsooli and Lin^51^). The Federal Emergency Management Agency (FEMA) used the model to update the coastal inundation maps^52^. The U.S. Army Corps of Engineers (USACE) utilizes the model in its high profile projects^53^. The model is also used in state-of-the-art flood forecasting systems, e.g., the Coastal Emergency Risks Assessment (CERA) system, which is developed to forecast hurricane flooding along the U.S. East and Gulf Coasts (https://coastalrisk.live/). In the coupled approach, ADCIRC and SWAN share the same unstructured finite element mesh. ADCIRC interpolates the wind field over the computational vertices spatially and temporally to calculate water levels and currents. Using ADCIRC’s wind field, water level, and currents data, SWAN calculates wind-generated water wave spectrum. The radiation stress due to breaking waves is then passed to ADCIRC to predict water levels and currents.

**Figure 1:**
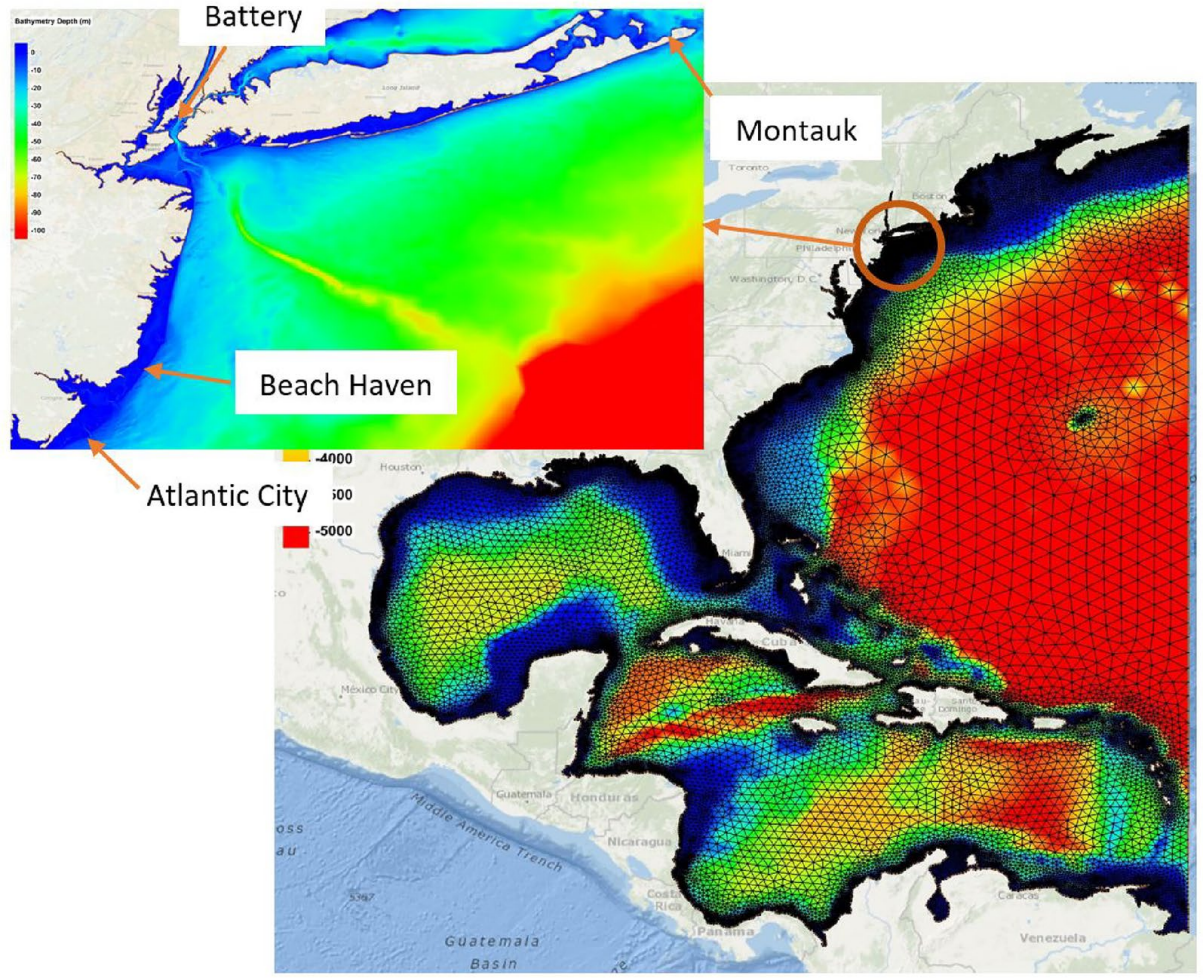
Computational mesh of the ADCIRC + SWAN model used in the present study. The study sites considered in this study are noted in the inset of the figure. The figure was generated using the AQUAVEO-SMS 13.1 software (https://www.aquaveo.com/software/sms-surface-water-modeling-system-introduction).

Figure 1 shows the two dimensional computational mesh developed by a study from FEMA’s Region II office^52^. The mesh covers the Western North Atlantic ocean with longitudes between $98^{\circ }$ and $60^{\circ }$ W, and latitudes between $8^{\circ }$ and $46^{\circ }$ N. The spatial resolution in the shallow nearshore zone (depth less than 8 m) is about 80 m and gradually increases to eight kilometers at depth 80 m. In deep water, the spatial resolution reaches 100 km. The computational time step and horizontal eddy viscosity are set to 3 s and 50 m$^2$s$^{-1}$, respectively. ADCIRC-2DDI accounts for the Coriolis force in the momentum equation to approximate the effect of planetary rotation on the storm surge. The bed roughness values (Manning’s n) coefficient varies spatially based on the land cover data^54^. The same roughness values are used in the wave model. The wind drag coefficient used in SWAN model is calculated using the method of Powel et al.^55^ with a cap of 0.0025. The computational time step in SWAN model is set to 15 min, which is also the coupling interval to the hydrodynamic model.

FEMA calibrated and validated the 2D ADCIRC + SWAN model output, i.e. maximum water elevation, using six historical tropical and extratropical events on the Northeast Coast^54^. The modeled peak water levels were compared against 218 high water mark readings from the six storms provided by the U.S. Army Corps of Engineers, and the NY Sea Grant publication Storm Surge^56^. The ADCIRC + SWAN model performance is considered to be acceptable if at least $70\%$ of the comparisons for each storm shows a difference between the modeled and measured peak water levels less than 0.46 m. The validation results show that more than $75\%$ of the comparisons for four storms, and more than $60\%$ of the comparisons of the other two storms have peak water level differences that are less than 0.46 m. Also, the validation shows that the absolute average difference ranges between 0.25 and 0.39 m with an average value of 0.32 m. Given the uncertainties in hindcasting the storms including, data collection, and meteorological and topographic data, FEMA considers the model as capable to simulate both tropical and extratropical storm events. The mesh was also used by the New York City Panel on Climate Change (NPCC2) for modeling NYC coastal flooding^57^, and in other studies^58–61^.

In this study, we focus on four study sites, namely Battery, and Montauk in New York (NY), which respectively cover the New York Harbor (NYH) and the entrance of long island sound, and Atlantic City and Beach Haven West in New Jersey (NJ), which respectively represent the open coast and back bay of NJ shoreline. The locations of the four stations are noted in the inset of Fig. 1.

#### Storm scenarios

The synthetic TCs used in this study are based on the TC data sets from Marsooli et al.^62^. Each data set contains thousands of TCs generated by the statistical/deterministic TC model of Emanuel et al.^63^ for the Atlantic basin. The TC model generates synthetic TCs for specific large-scale atmospheric and oceanic conditions based on observations or climate models. The chosen data sets included 27,800 synthetic TCs. Of these, 23, 412 impacted the geographical area covered in this study. To determine the low-probability high-consequence (impactful storm surge) at an acceptable computational cost, we firstly simulated all 23, 412 TCs using ADCIRC only with relatively low spatial and temporal resolutions. From these simulations, we identified 1300 TCs as low-probability events that caused peak storm surge heights more than 0.5 m at the considered study sites. To complete the training data set, we randomly selected another 9000 TCs and added them to the 1300 TCs. High-fidelity ADCIRC + SWAN simulations of the 10,300 TCs were then performed to determine the peak storm surge height. The generated data set from these simulations was then used to train, validate and test the ML models.

**Figure 2:**
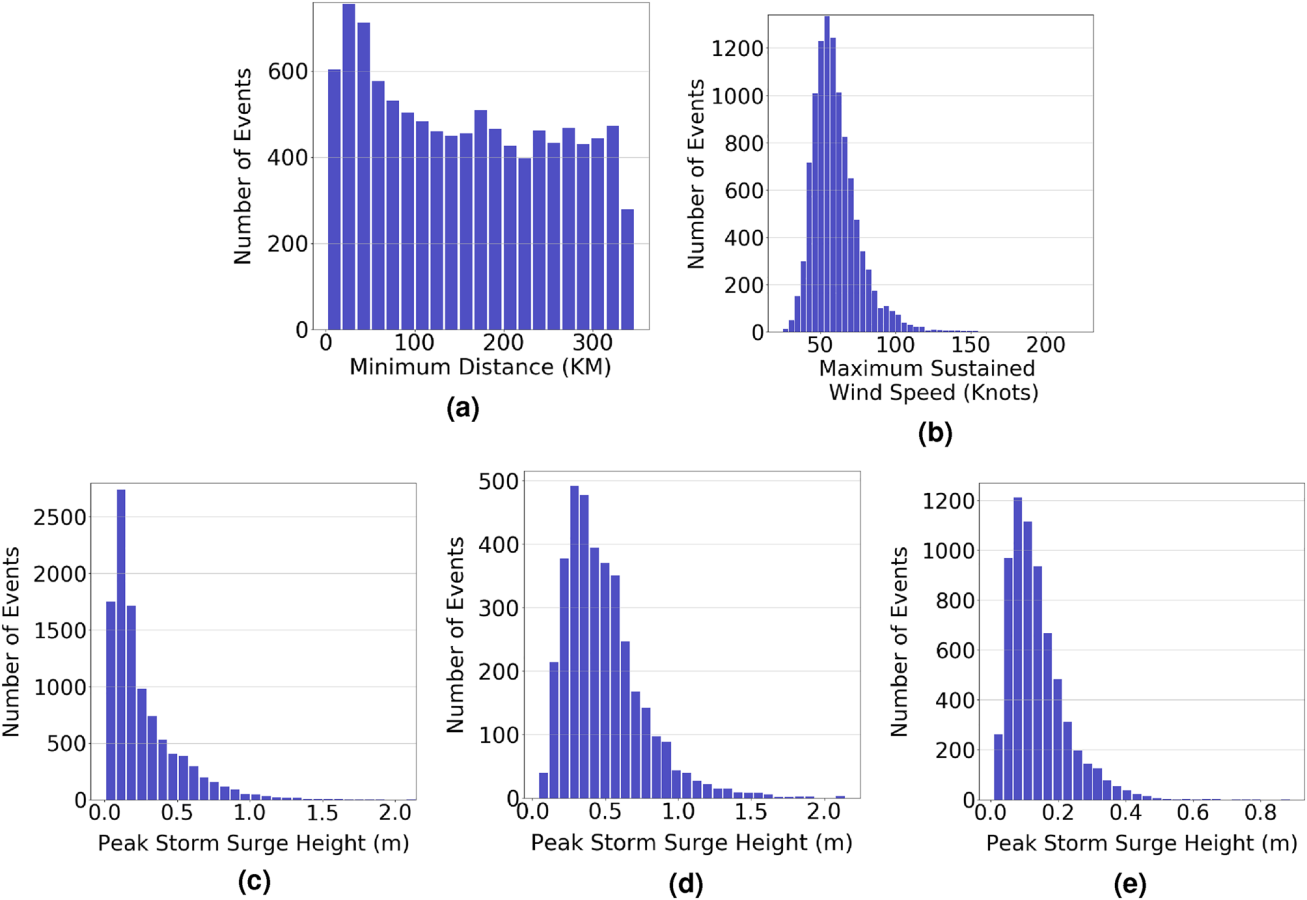
Histograms of the (**a**) minimum distance between TC eye and the study site, (**b**) sustained maximum wind speed when the TC is closest to the study site for the full data set, peak storm surge height for (**c**) the full data set, (**d**) TCs that pass within 100 km (DS-1), and (**e**) TCs that pass outside the 100 km region (DS-2). All histograms are for the Battery station.

**Figure 3:**
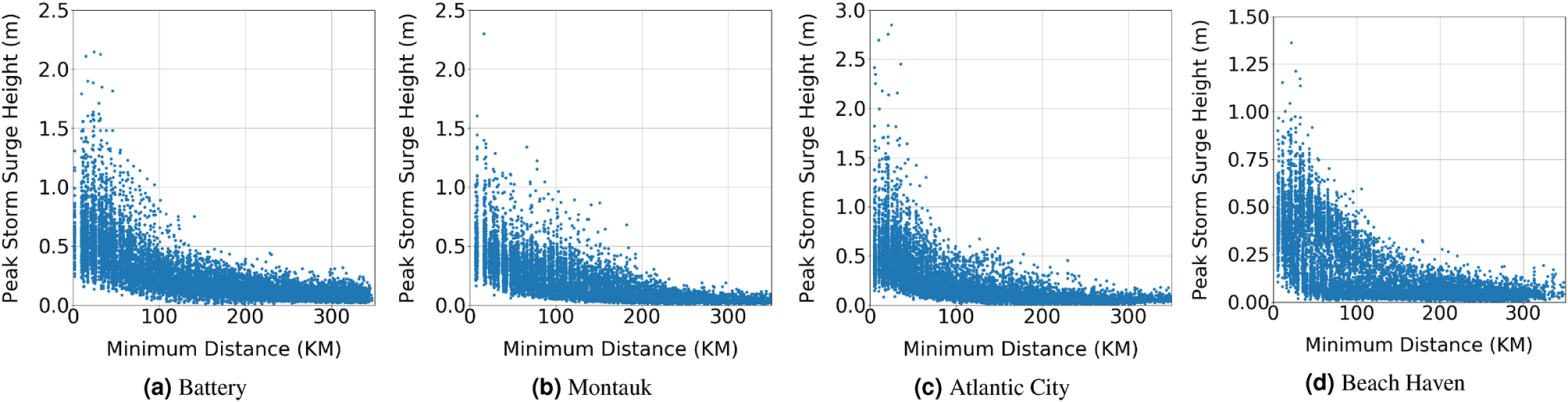
Scatter plots between the peak storm surge height calculated using the high-fidelity ADCIRC + SWAN simulations and the minimum distance between the TC eye and the study sites.

Measuring the TC impact at a study site requires a definition of its intensity which is represented by its maximum sustained wind speed ($V_{max}$). Because this speed varies as the TC tracks along a specific path, we define the TC intensity by its maximum sustained wind speed when it is closest to a study site. Based on this definition, histogram of the maximum sustained wind speed for the Battery station, based on the 10, 300 TCs, is presented in Fig. 2b. The plot shows that the maximum sustained wind speed follows a normal distribution with a mean value of 60 Knots. Figure 2a shows the histogram of the minimum distance between the TC eye and the Battery ($d_{min}$) of the whole data set. The plot shows that the minimum distance is uniformly distributed between 0 and 350 km. The histogram of the corresponding peak storm surge height calculated by ADCIRC + SWAN is presented in Fig. 2c. The plot shows that the peak storm surge height follows a right skewed distribution with a mean value of 0.26 m and a median value of 0.17 m. Only 1500 TCs, i.e. about $15\%$ of the simulated TCs, generated a peak storm surge height more than 0.5 m. Clearly, the data set is imbalanced and biased more to the smaller peak storm surge heights, which biases the trained ML model when considered as one set. The bias can be reduced by splitting the data set into smaller ones that are less biased. We used $d_{min}$ to split the data set as it has the highest impact on the peak storm surge height, as shown in the following section. Figure 3 shows a scatter plot between the minimum distance and corresponding peak storm surge height, which is used to choose the threshold value of $d_{min}$. As shown in the plots, the peak storm surge height decreases as $d_{min}$ increases to approach an asymptote with a value less than 0.5 m beyond $d_{min} = 100$ km. Also, the plots show that for $d_{min}$ more than 100 km, the peak storm surge height dropped to less than 1 m at the Battery and Montauk, and 0.5 m in Atlantic City and Beach Haven. This also can be seen from the histograms of peak storm surge height for TCs that pass within and outside a radius of 100 km from the Battery station as shown in Fig. 2d,e, respectively. The 3650 TCs that pass within a radius of 100 km have a maximum peak storm surge height of 2.14 m, while the 6650 TCs that pass outside a radius of 100 km have a maximum peak storm surge height of 0.89 m. The histogram plot Fig. 2d also shows that almost $50\%$ of the TCs that pass within the 100 km radius generate a storm surge peak larger than 0.5 m. Based on these histograms, we divide the data set into two smaller ones, namely DS-1 and DS-2 that respectively include TCs that pass within and outside a radius of 100 km from the study site to realize balanced data sets for training the ML models.

### Data analysis and pre-processing

#### TC parameters

Different linear and nonlinear ML models are implemented to predict the peak storm surge height from the TC parameters, including intensity and track. In the following, the subscript *i* = 6, 0, and $-6$, is used to denote the values of these parameters respectively at 6-h post, 0-, and 6-h prior to the time of the closest TC location to the study site. Six parameters are used to describe the TC at each time step. Its intensity is represented by the maximum sustained wind speed (${V_{max}}_i$), its size is represented by the radius of maximum wind (${R_{max}}_i$), and its track is represented by four values, namely the upper (${lat_{upper}}_i$) and lower (${lat_{lower}}_i$) latitudinal distance, and the right (${lon_{right}}_i$) and left (${lon_{left}}_i$) longitudinal distances. If the eye of the TC is on the right side (above) the point of interest, then the left longitudinal (lower latitudinal) distance is zero and the right longitudinal (upper latitudinal) distance is equal to the distance with a negative sign. If the TC’s eye is on the left side (below) the point of interest, then the right longitudinal (upper latitudinal) distance is zero and the left longitudinal (lower latitudinal) distance is equal to the distance with positive sign. An additional parameter is used to identify the TC track which is the minimum absolute distance between the TC eye and the study site ($d_{min}$). Thus, a total of 19 features ($=$ six TC parameters $\times$ three time steps $+$ the minimum distance) are used as the ML models features.

#### Feature selection

Feature selection is conducted by calculating the correlation and degree of dependence of the peak storm surge height ($\eta _{TC}$) on the 19 features ($X_i$) using the correlation coefficient (*R*) and mutual information (*MI*) values^64^ that are respectively defined as:

1 $$\begin{aligned} R= & {} \frac{cov\left( \eta _{TC},X_i\right) }{\sigma _{\eta _{TC}} \sigma _{X_i}} \end{aligned}$$

2 $$\begin{aligned} MI= & {} D_{KL} \left[ P\left( \eta _{TC},X_i\right) || P\left( \eta _{TC}\right) P\left( X_i\right) \right] \end{aligned}$$

where, *cov*(., .) and $\sigma _.$ are, respectively, the covariance and standard deviation, $D_{KL}$ is the Kullback-Leibler distance between two probability distributions, $P\left( \eta _{TC},X_i\right)$ is the joint distribution of the peak storm surge height and each TC parameter, and $P\left( \eta _{TC}\right)$ and $P\left( X_i\right)$ are the corresponding marginal distributions. The linear correlation between peak storm surge height and TC parameters *R* has either a positive or negative value depending on whether they are positively or negatively correlated, respectively. The *MI* shows the nonlinear correlation, which is always positive and ranges from 0 to 1 signifying no and high dependence on the TC parameter, respectively.

**Figure 4:**
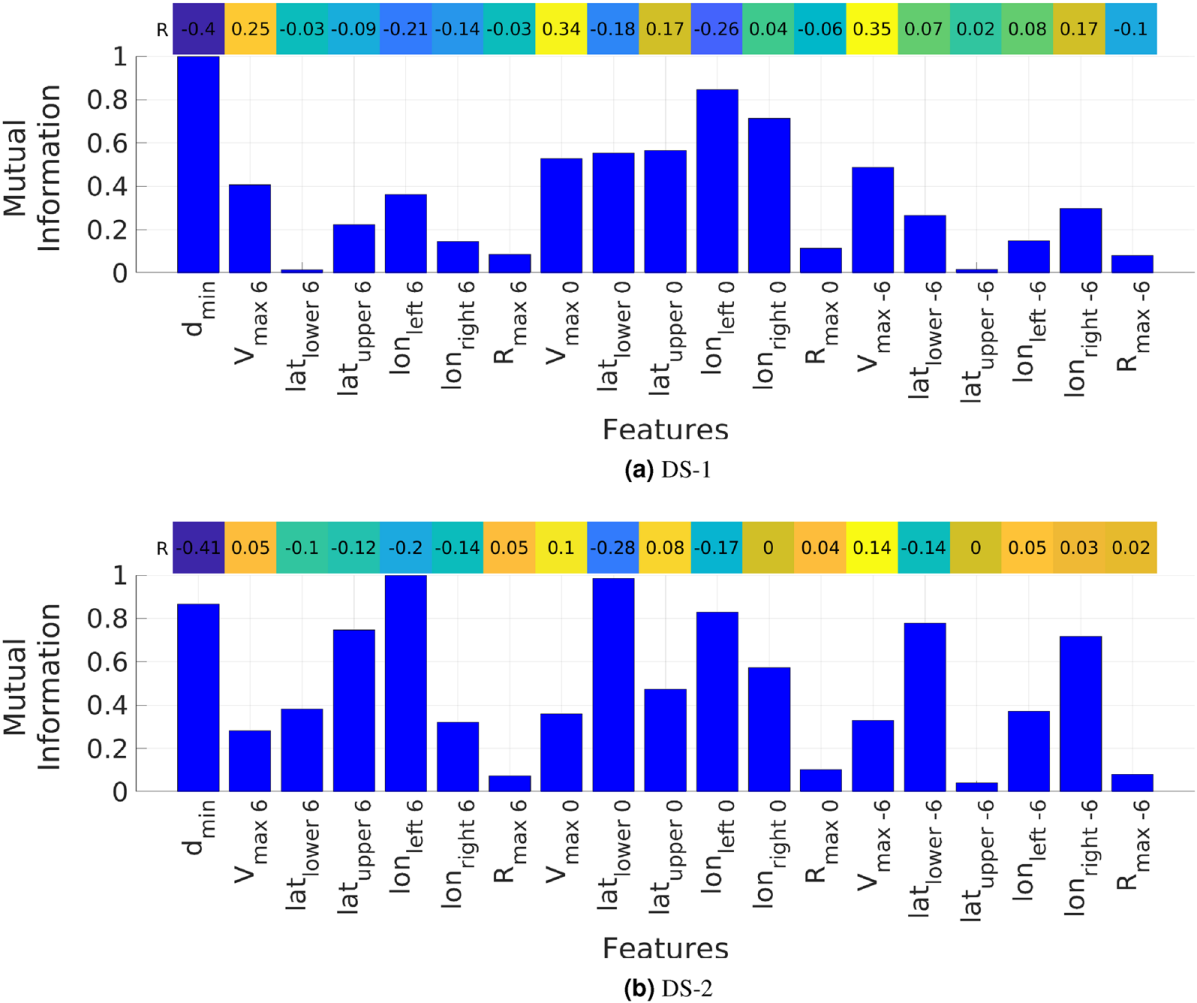
Correlation coefficients (color bar) and relative mutual information values (bar plot) between the peak storm surge height and the 19 features of the two data sets.

The correlation coefficient and relative mutual information values between the peak storm surge height (calculated by ADCIRC + SWAN) and the 19 features for the two data sets are presented in Fig. 4. The color bars show the correlation coefficient and the bar plots shows the mutual information values. In both data sets, the maximum correlation of the peak surge height is $d_{min}$. Figure 3 shows the high dependence of peak storm surge height to $d_{min}$ where the surge decreases as $d_{min}$ increases. Nearly the same level of correlation is noted between the peak storm surge height and ${V_{max}}_i$ in data set DS-1. The highest level of mutual information in data set DS-1 is noted to be between the peak surge height and $d_{min}$. In data set DS-2, high values of mutual information are noted between the peak surge height and $d_{min}$, ${lat_{upper}}_i$, ${lat_{lower}}_i$, ${lon_{right}}_i$, and ${lon_{left}}_i$. The effect of $V_{max}$ on the peak storm surge height decreases as $d_{min}$ increases^65^, which explains the high degree of dependence of this peak on the TC track. Therefore, stronger but farther hurricanes are less effective than weaker and closer ones.

In addition to identifying the parameters having the highest correlation with the peak storm surge height, the above two coefficients are used to remove features based on low correlation values. For that, we firstly neglected the features that have lowest *R* and *MI*. Then, we tested the different ML models using different combinations of TC parameters. We ended up with a total of 13 different features for each of the two data sets. The three $R_{max}$, ${lon_{left}}_{-6}$ and ${lat_{upper}}_{-6}$ features were removed from both sets, while ${lat_{lower}}_6$ and ${lon_{right}}_0$ were removed from the features of DS-1 and DS-2, respectively.

### ML models and quantification

ML algorithms build a model based on training data set in order to make predictions without being explicitly programmed to do so. The algorithms are categorized as supervised when the training data set has labeled input and output data, and unsupervised when the data set is not labeled. In this study we adopt supervised algorithms only as the data set is labeled. Seven linear and non-linear ML models are implemented and tested. The used ML models are Ridge Regression (RR), Support Vector Regressor (SVR), Decision Tree Regressor (DTR), Random Forest Regressor (RFR), Extra Trees Regressor (ETR), Gradient Boosted Decision Tree Regressor (GBDTR) and Adaptive Boost (AdaBoost) Regressor. Each of these algorithms has its own hyperparameters that should be optimized.

**Ridge regression** is a variation of the ordinary least squares (OLS) method which expects that the target value is a linear combination of the features. The OLS aims to minimize objective function function

3 $$\begin{aligned} \min _w ||Xw-y||_2^2 \end{aligned}$$

where *X* is the input feature, *y* is the target, and *w* is the weight of the linear combination. Highly correlated features (collinearity) make the OLS more sensitive to random errors. The RR addresses this issue by imposing a penalty on the weight values by adding L2-norm of the weights multiplied by the penalty parameter ($\alpha$) to the loss function.

4 $$\begin{aligned} \min _w ||Xw-y||_2^2 + \alpha ||w||_2^2 \end{aligned}$$

The penalty parameter controls the amount of shrinkage of the weights. The larger the value of $\alpha$, the smaller the weights are and thus more robust to collinearity. The hyperparameters that will be tuned to get the best results are $\alpha$, the solver to use in computational routines, and the stopping criteria defined by the tolerance of error.

**Support vector regressor** main objective is to minimize L2-norm of the coefficient vector. The optimization problem, Eq. (5), is constrained such that the absolute error between the predicted and true values is less than or equal to a specified margin ($\varepsilon > 0$).

5 $$\begin{aligned} \begin{aligned} \min _w \quad&||w||_2^2 \\ s.t. \quad&|y-wX| \le \varepsilon \end{aligned} \end{aligned}$$

This constrained optimization problem does not account for the data points outside the margin $\varepsilon$. Thus, an L$-1$ penalized term is added to the objective function to include extra points, equation 6.

6 $$\begin{aligned} \begin{aligned} \min _w \quad&||w||_2^2 + C \sum _{i=1}^n \zeta _i \\ s.t. \quad&|y-wX| \le \varepsilon + |\zeta | \end{aligned} \end{aligned}$$

where $C > 0$ is the penalty term, and $\zeta$ is a slack variable defined as the deviation of data point from the margin. As *C* increases, the tolerance for points outside the interval $\varepsilon$ increases. Thus, the hyperparameters that will be tuned to get the best results are *C*, and $\varepsilon$

**Decision tree regressor** is a non-parameteric supervised learning method. They are generated in a recursive process. A decision tree is composed of root nodes, internal nodes, and leaf nodes where the nodes are connected by branches. The root node is the topmost node that has the complete data set, each internal node denotes a test, each branch represents a test attribute, and each leaf node corresponds to a result. The path from the root node to each leaf node corresponds to a sequence of test judgments which is based on the Gini index. The hyperparameters that will be tuned to get the best results are the maximum depth of the tree (max depth) and minimum number of samples required on a leaf node (min samples leaf).

**Random forest and extra trees regressors** are parallel ensemble methods of the decision trees. They are based on training multiples of weak learners, then the outputs are averaged to obtain the final predicted output. The main differences between the random forest and extra trees are that the RFR subsamples the input data with replacement (bootstrap) while the ETR uses the whole data set. Also, the test judgements in RFR is based on optimum split while it is random in ETR . The hyperparameters that will be tuned to get the best results are the maximum depth of the tree (max depth), the minimum number of samples required on a leaf node (min samples leaf), and the number of weak learners (n estimators).

**Adaptive boost and gradient boosted decision tree regressors** are based on a boosting technique, i.e. a sequential ensemble method. Initially, a weak learner (base estimator) is trained using the whole training data set. This data set is then adjusted based on the prediction of the weak learner, so that it gives more attention to the incorrectly predicted samples by the previous weak learner. This cycle is repeated until it reaches the specified number of weak learners (n estimator). Finally, all weak learners are weighted and combined. AdaBoost assigns weights to every data point in the data set. The weights of wrongly predicted values in the first weak learner will increase in the following ones. The base estimator in the AdaBoost can be any ML algorithm. On the other hand, the GBDTR uses regression decision trees as their base estimators. It uses the residual of the current regression tree as the input to the consecutive tree. Thus, each regression tree learns the conclusions and residuals of all previous trees, and fits a current residual regression tree. Both AdaBoost and GBDTR use the weighted sum of the weak learners to get the final result. The hyperparameters of the AdaBoost are the base estimator, learning rate (LR), the number of weak learners (n estimators), and the loss function, while those of the GBDTR are the learning rate (LR), number of weak learners (n estimators), and maximum depth of the tree (max depth).

#### Performance metrics

The performance of the different models was evaluated by comparing the peak storm surge height as predicted from the ML models ($\eta _p$) to those calculated from ADCIRC + SWAN models ($\eta _a$). Different metrics have been used in the literature to evaluate the performance of ML models with respect to true data. Here, we adopt the correlation coefficient (*R*), Eq. (1), and coefficient of determination (*R*2) defined as

7 $$\begin{aligned} R2 = 1-\frac{\sum _{i=1}^{N} ({\eta _a}_i-{\eta _p}_i)^2}{\sum _{i=1}^{N} ({\eta _a}_i-{{\overline{\eta }}_a}_i)^2} \end{aligned}$$

where *N* is the size of the data set, and ${\overline{\eta }}_a$ is the average value of ${\eta }_a$. The absolute values of *R* and *R*2 range between zero and one respectively signifying no and perfect match. We also use Relative Absolute Error and Mean Relative Absolute Error (*RAE*, *MRAE*) defined respectively as

8 $$\begin{aligned} RAE= & {} \left| \frac{\eta _a - \eta _p}{\eta _a}\right| *100\% \end{aligned}$$

9 $$\begin{aligned} MRAE= & {} \frac{1}{N} \sum _{i=1}^{N} RAE_i \end{aligned}$$

to measure the performance of the ML models. Zero values of these errors indicate the perfect match. Lastly, we use the Root Mean Square Error (*RMSE*) defined as

10 $$\begin{aligned} RMSE = \sqrt{\frac{1}{N} \sum _{i=1}^{N} \left( {\eta _a}_i - {\eta _p}_i \right) ^2} \end{aligned}$$

with a zero value indicating perfect matching.

#### Hyper-parameters tuning

In developing these models, $60\%$ of the DS-1 and DS-2 data sets were used for training while $20\%$ were used for validation. The rest were used for testing the models’ performance. The training was performed using scikit-learn library on Python^66^. The used hyper-parameters of each ML model, presented in Table 1, were tuned using the cross-validation grid search method, by trying all possible combinations between the hyper-parameters and by getting the best performing configuration for training.

**Table 1:** Tuned hyper-parameters of the ML models used to train the two data sets, DS-1 and DS-2.

RR			SVR			DTR			RFR		
Parameters	DS-1	DS-2	Parameters	DS-1	DS-2	Parameters	DS-1	DS-2	Parameters	DS-1	DS-2
$\alpha$ solver tolerance	0.1 lsqr 1e-4	0.1 lsqr 1e–3	C $\varepsilon$	75 0.2	65 0.03	Max depth min samples leaf	24 7	13 11	Max depth min samples leaf n estimators	20 2 180	35 2 250

ETR			GBDTR			AdaBoost regressor					
Parameters	DS-1	DS-2	Parameters	DS-1	DS-2	Parameters	DS-1		DS-2		
Max depth min samples leaf n estimators	21 2 130	18 2 70	n estimators LR max depth	200 0.1 5	270 0.1 6	Base estimator LR loss function n estimator	SVR (C = 90, epsilon = 0.09) 0.09 exponential 15		SVR (C = 65, epsilon = 0.03) 0.05 exponential 50		

## Results

### ML results

The performance metrics, as defined above, of the seven models are presented in Table 2 using the test data sets. Decision Tree models are extremely sensitive to small changes in data, easy to overfit, and are not able to deal with collinearity, i.e. when two variables represent the same thing. The Ridge Regression model trades variance for bias, i.e. the output has a low variance but is biased, which causes large errors in the case of small number of features with respect to the number of samples. Thus, based on the numbers in Table 2, the Ridge Regression and Decision Tree models have consistently lower correlation and determination coefficients and higher *MRAE* and *RMSE* values. On the other hand, the SVR is robust to outliers, does not suffer from overfitting, and is not affected by small changes in the data. The AdaBoost model is less prone to overfitting, and utilizes the weighted average of multiple weak learners using a base estimator to yield a robust result. Thus, by using SVR as the base estimator for AdaBoost, we combine the advantages of the two models. Therefore, SVR and AdaBoost models yield the highest correlation and determination coefficients and lowest *MRAE* and *RMSE* values. For the same model, slight differences in the performance metrics are noted among the different locations for the two data sets.

**Table 2:** Performance metrics of the seven ML models at the four study sites using the test data set.

Machine learning models	Data set	Battery				Montauk				Atlantic City				Beach Haven			
		R	R2	MRAE (%)	RMSE (cm)	R	R2	MRAE (%)	RMSE (cm)	R	R2	MRAE (%)	RMSE (cm)	R	R2	MRAE (%)	RMSE (cm)
Ridge regression	DS-1	0.82	0.68	28.7	15.1	0.86	0.74	30.1	10.2	0.79	0.62	43.8	17	0.83	0.69	71.2	10.9
	DS-2	0.74	0.55	44.3	5.7	0.73	0.54	82.3	6.9	0.65	0.43	64.8	5.6	0.68	0.46	68.8	4.7
SVR	DS-1	0.94	0.88	17	9.1	0.95	0.91	15.9	6.1	0.96	0.91	19.3	8.2	0.97	0.93	28.2	5
	DS-2	0.89	0.79	25	3.9	0.96	0.92	24.4	2.8	0.92	0.85	24.7	2.9	0.93	0.87	25.2	2.3
Decision tree	DS-1	0.88	0.77	23.6	12.6	0.9	0.82	18.8	8.6	0.85	0.72	25	14.7	0.94	0.88	25.8	6.8
	DS-2	0.82	0.66	32.9	5	0.92	0.84	28.8	3.9	0.85	0.73	30.1	3.8	0.89	0.79	28.7	3
Random forest	DS-1	0.92	0.85	19.4	10.4	0.94	0.88	15.3	6.9	0.92	0.85	20.3	10.6	0.96	0.92	21.1	5.4
	DS-2	0.89	0.79	26.5	3.9	0.95	0.91	24.1	3	0.91	0.83	25.7	3	0.93	0.85	25.3	2.5
Extra tree	DS-1	0.93	0.87	18.2	9.7	0.94	0.89	15.4	6.7	0.94	0.88	19.4	9.6	0.96	0.93	21.1	5.2
	DS-2	0.9	0.8	26.2	3.8	0.96	0.92	23.9	2.8	0.92	0.85	25.7	2.9	0.93	0.87	26.4	2.3
Gradient boost	DS-1	0.93	0.87	17.4	9.5	0.94	0.88	15.1	6.9	0.94	0.88	18.6	9.5	0.96	0.93	22.1	5.2
	DS-2	0.88	0.77	27.4	4	0.96	0.94	22.9	2.7	0.92	0.84	25.7	2.9	0.92	0.85	24.8	2.5
Adaptive boost	DS-1	0.94	0.89	16.4	9	0.95	0.91	14.4	6.1	0.95	0.92	17.8	8	0.97	0.94	20.3	4.8
	DS-2	0.89	0.8	25.2	3.8	0.96	0.92	24.3	2.8	0.92	0.85	25.1	2.8	0.93	0.87	25.7	2.3

**Figure 5:**
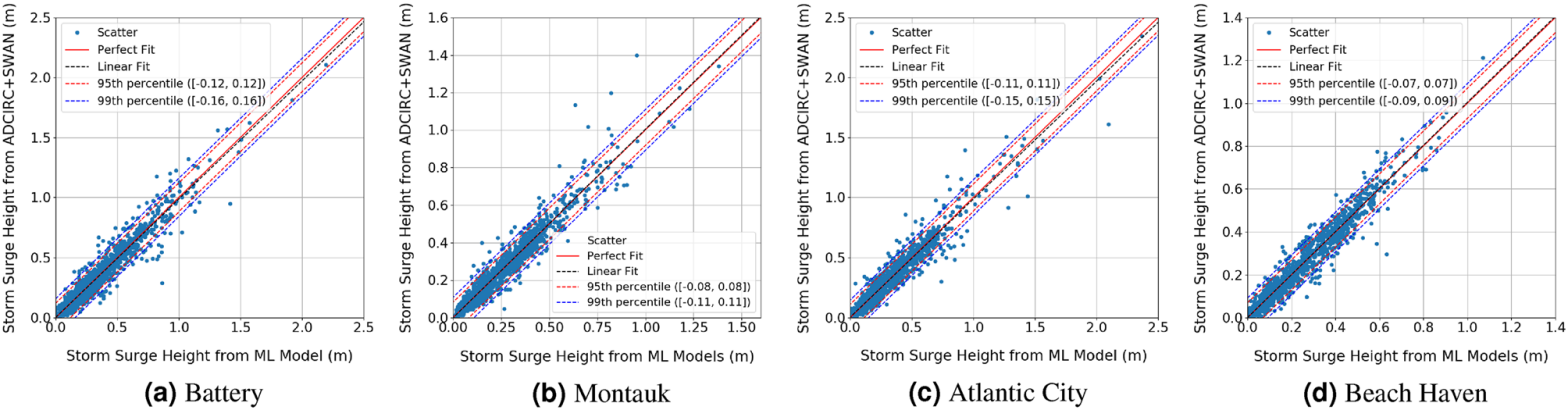
Scatter plots between the peak storm surge height calculated using the high-fidelity ADCIRC + SWAN simulations and those predicted from ML models at the four study sites.

**Figure 6:**
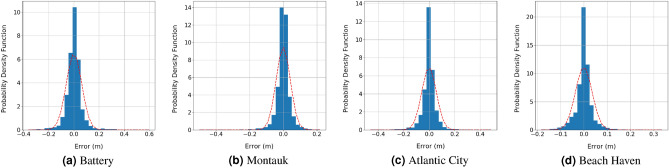
Histograms of the error between peak storm surge heights determined by the high-fidelity ADCIRC + SWAN simulations and those predicted from ML models at the four study sites.

Figure 5 shows the scatter plots of the peak storm surge heights calculated using ADCIRC + SWAN models with those predicted from the ML models of the test data set of the (DS-1, and DS-2) data sets combined. Ideally, the scatter points should be close to the regressed diagonal line that represents a perfect fit. The linear fit of the scattered points should also coincide with the perfect fit line with slope 1. The slopes of the linear fit of the test data at Battery, Montauk, Atlantic City, and Beach Haven West are 0.98, 1, 0.98, and 1, respectively. This indicates a high level of agreement between the ADCIRC + SWAN results and ML models predictions. Further evidence of the goodness of the ML models predictions is noted from the histograms and corresponding normal distribution fits of the error, defined by the difference between the storm surge height calculated using ADCIRC + SWAN and predicted from ML models for the two test data sets, which are presented in Fig. 6. The mean, and standard deviation of the error at the four stations are about 0 and 5 cm, respectively. The reference lines, represented by dashed lines, in Fig. 5 indicates the 95-th and 99-th percentiles calculated from the normal distribution fits of the errors in Fig. 6, on average 64 out of 2072 storms are outside the 99-th percentile range and 108 are outside the 95-th percentile range. These results show that, to great extent, the peak storm surge heights predicted from the ML models match those simulated by ADCIRC + SWAN model.

### Probabilistic flood hazard assessment

#### Statistical model

The probabilistic flood hazard assessment is presented in terms of peak storm surge height at the four selected study sites. Assuming that the TC’s arrival is distributed as a stationary Poisson process, the return period (*T*) of peak storm surge height ($\eta _{TC}$) exceeding a given threshold (*h*) is given by^67^

11 $$\begin{aligned} T = \frac{1}{F \left( 1- P \left[ \eta _{TC} \le h \right] \right) } \end{aligned}$$

where *F* is the TC annual frequency, and $P \left[ \eta _{TC} \le h \right]$ is the cumulative probability distribution (CDF). The distribution of the peak storm surge heights due to TCs is characterized by long tail which is modeled using the Generalized Pareto Distribution (GPD)^68^

12 $$\begin{aligned} H(y) = 1 - \left[ 1 + \frac{\zeta *(y-u)}{\sigma _u} \right] ^{-1/ \zeta } \end{aligned}$$

where $H(y) = P[y>u]$ is the CDF of the GPD, and *u* is the GPD threshold value which is selected by try and error so that the modeled CDF well represents the data points. The parameters $\sigma _u$ and $\zeta$ are the controlling parameters of the scale and shape of the GPD distribution, respectively. Both parameters are statistically estimated using the Maximum Likelihood method.

#### Return periods results

**Figure 7:**
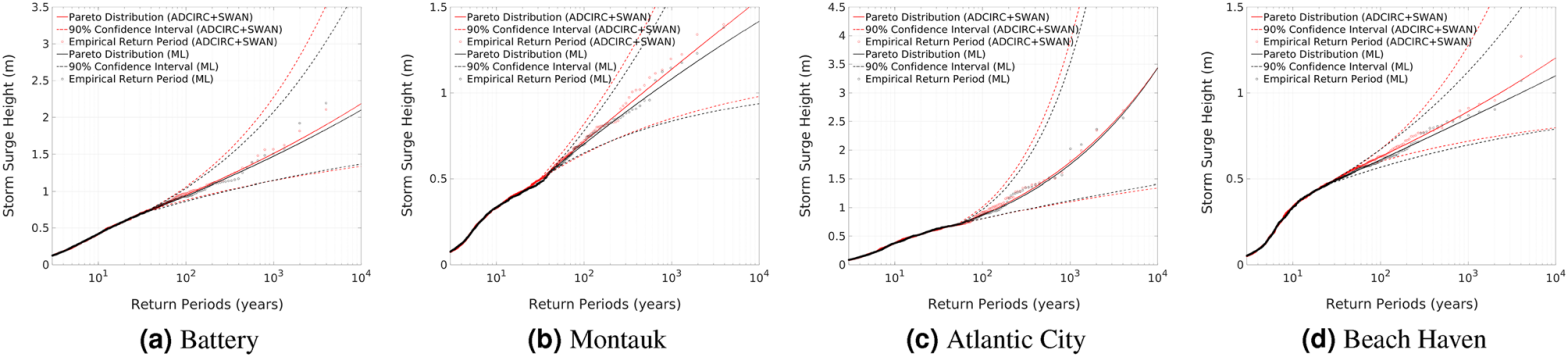
Comparison of return period curves with confidence intervals at the four study sites using peak storm surge generated by ADCIRC + SWAN and ML models.

Figure 7 shows the empirical and Pareto distribution fits of the peak storm surge heights return period along with the 90-th percentile confidence interval for the four study sites. Using the test data set, plots from both ADCIRC + SWAN simulated and ML predicted results are presented. A frequency of 0.52 storm per year is assumed for both ADCIRC + SWAN and ML results. The used Pareto threshold values, and the *RAE* between the predicted 10-, 100-, 1000-, and 4000-year return periods using ADCIRC + SWAN and ML results are presented in Table 3. The maximum *RAE* is less than $0.7\%$ for the 10-year return period, $5\%$ for the 100- and 1000-year return periods, and $7\%$ for the 4000-year return period. Also, the *RMSE* between the Pareto distribution fits using ADCIRC + SWAN and ML results for the four study sites is less than 7.5 cm. These results indicate that return period curves generated from ML predictions match very well those generated from ADCIRC + SWAN simulations but at a fraction of the computational time and resources.

**Table 3:** Comparison of Pareto thresholds and return period results at the four stations using ADCIRC + SWAN and ML results.

Station name	Pareto threshold		*RAE* (%)				RMSE (cm)
	ADCIRC	ML	10	100	1000	4000	
Battery	0.7338	0.688	0.3	1.6	2.5	4	4.8
Montauk	0.489	0.539	0.7	1.9	5	6.9	7.3
Atlantic City	0.694	0.709	0.6	1.6	2.2	1.1	3.5
Beach Haven	0.548	0.455	0.6	3	4.6	6.8	5.4

## Conclusions

We demonstrated the usefulness of artificial intelligence in reducing the computational burden of predicting high-consequence low-probability (up to 4000-year return period) storm surge from tropical storms. Given the geographic boundaries of TC development in terms of their track and variations in their strength, it was necessary to take into consideration the impact of these variations on model training. Seven linear and non-linear ML models were implemented and tested. Their hyperparameters were optimized. Model was trained using simulations performed with the coupled ADvanced CIRCulation and Simulating WAves Nearshore (ADCIRC + SWAN) models of peak storm surge heights in the metropolitan New York area. The input to the ML models are TC parameters that included maximum sustained wind speed, upper and lower latitudinal, right and left longitudinal distances, and minimum distance between our point of interest and the TC eye, at three time steps, namely the moment of minimum distance and 6 h before and after this moment. The analysis showed the need to divide the data set to overcome an imbalance problem in the total data set. Evaluation of performance metrics showed that Support Vector Regression and Adaptive Boosting models performed better than others in terms of predicting peak surge heights because they are robust to outliers, less prone to overfitting, and not affected by small changes in the data. These predictions yield return period curves that matched very well those generated from high-fidelity but computationally expensive ADCIRC + SWAN simulations. The results demonstrated the efficiency of surrogate models in predicting return periods when compared with ADCIRC + SWAN simulations.

## Data Availability

The data that support the findings of this study are available from the corresponding author, M.A., upon reasonable request.
